# Functional Traits in Lichen Ecology: A Review of Challenge and Opportunity

**DOI:** 10.3390/microorganisms9040766

**Published:** 2021-04-06

**Authors:** Christopher J. Ellis, Johan Asplund, Renato Benesperi, Cristina Branquinho, Luca Di Nuzzo, Pilar Hurtado, Isabel Martínez, Paula Matos, Juri Nascimbene, Pedro Pinho, María Prieto, Bernardo Rocha, Clara Rodríguez-Arribas, Holger Thüs, Paolo Giordani

**Affiliations:** 1 Royal Botanic Garden Edinburgh, 20A Inverleith Row, Edinburgh EH3 5LR, UK; 2 Faculty of Environmental Sciences and Natural Resource Management, Norwegian University of Life Sciences, 5003 NO-1432 Ås, Norway; johan.asplund@nmbu.no; 3 Dipartimento di Biologia, Università di Firenze, Via la Pira, 450121 Florence, Italy; renato.benesperi@unifi.it (R.B.); luca.dinuzzo@unifi.it (L.D.N.); 4 Centre for Ecology, Evolution and Environmental Changes (cE3c), Faculdade de Ciências, Universidade de Lisboa, Campo Grande, C2, Piso 5, 1749-016 Lisboa, Portugal; cmbranquinho@fc.ul.pt (C.B.); paplopes@fc.ul.pt (P.P.); brerocha@fc.ul.pt (B.R.); 5 Área de Biodiversidad y Conservación, Departamento de Biología, Geología, Física y Química Inorgánica, ESCET, Universidad Rey Juan Carlos, 28933 Móstoles, Spain; pilar.hurtado@urjc.es (P.H.); isabel.martinez@urjc.es (I.M.); maría.prieto@urjc.es (M.P.); clara.rodriguez@urjc.es (C.R.-A.); 6 Departamento de Biología (Botánica), Universidad Autónoma de Madrid, c/Darwin, 2, 28049 Madrid, Spain; 7 MARE—Marine and Environmental Sciences Centre, Faculdade de Ciências, Universidade de Lisboa, Campo Grande, 1749-016 Lisboa, Portugal; psmatos@fc.ul.pt; 8 Department of Biological, Geological and Environmental Sciences, Alma Mater Studiorum, University of Bologna, I-40126 Bologna, Italy; juri.nascimbene@unibo.it; 9 Botany Department, State Museum of Natural History Stuttgart, Rosenstein 1, 70191 Stuttgart, Germany; holger.thues@smns-bw.de; 10 DIFAR, University of Genova, Viale Cembrano, 4, I-16148 Genova, Italy; giordani@difar.unige.it

**Keywords:** ecosystem services, effect traits, functional ecology, lichenised-fungi, life-history strategy, response traits, scale, spatial, temporal

## Abstract

Community ecology has experienced a major transition, from a focus on patterns in taxonomic composition, to revealing the processes underlying community assembly through the analysis of species functional traits. The power of the functional trait approach is its generality, predictive capacity such as with respect to environmental change, and, through linkage of response and effect traits, the synthesis of community assembly with ecosystem function and services. Lichens are a potentially rich source of information about how traits govern community structure and function, thereby creating opportunity to better integrate lichens into ‘mainstream’ ecological studies, while lichen ecology and conservation can also benefit from using the trait approach as an investigative tool. This paper brings together a range of author perspectives to review the use of traits in lichenology, particularly with respect to European ecosystems from the Mediterranean to the Arctic-Alpine. It emphasizes the types of traits that lichenologists have used in their studies, both response and effect, the bundling of traits towards the evolution of life-history strategies, and the critical importance of scale (both spatial and temporal) in functional trait ecology.

## 1. Introduction

Focusing on groups such as vascular plants and freshwater invertebrates [1,2] the ecological approach to explaining biodiversity has undergone a major transition from the specific, taxonomically descriptive and pattern-based [3,4,5], towards the dynamically process-based, generalisable and predictive [6,7,8]. The logic behind this transition is the identification of functional traits (sensu [9]) as the measurable attributes of an organism’s phenotype that account for its ecological response to one or more biotic or abiotic, spatial or temporal gradients constituting the environment. Accordingly, the distributions, occurrence and abundances of lichen species, and their contributions to community structure, are explained by identifying non-random patterns of functional traits in space and time, allowing the subsequent inference of assembly rules [10,11].

Lichens have an important but still unfulfilled potential in completing this conceptual transition from pattern to process. For example, as the cohabitant combination of fungi and algae (and/or cyanobacteria) they lie within the realm of ‘microorganisms’ but are often of a size and longevity that is suited to practical investigation, and so, being amenable to standard ecological tools, they can open a window on species response and community assembly relevant to a wider spectrum of the Earth’s biodiversity. They are also the outcome of a complex evolutionary process involving the different symbiotic partners, and leading to a diversity of phenotypes that reflect, to varying degrees, adaptive radiation or convergent evolution, so providing the rich multivariate framework required to explore linkages between ecological fitness, environmental gradients and ecosystem function. Applying a trait-based approach to lichens has important practical benefits in conservation too; for example, it is the difference between a taxonomic list such as for lichen indicators observed to associate with ecological continuity [12,13,14,15], and understanding—with its increased confidence—the features of lichen biology that are mechanistically linked to old-growth conditions because of interactions with environmental constraints [16,17,18].

If functional traits can help us explain and understand the species response to the environment (see Types of Traits, below), they can also, by extension, invoke consequences for ecosystem processes (see Effect Traits, below). The multifaceted power of this functional trait approach, as applied to lichens, is explored in this review. There are three key points that we want to emphasise:

- First, the trait-based approach is integrative, since traits can be explored with respect to community composition and richness, explaining the response of species to the environment, but also the effect that species have within ecosystems, bridging therefore between the classic dichotomy of ‘Grinnelian’ and ‘Eltonian’ niche models (see Effect Traits, below).
- Second, it is an approach that links evolutionary biology—the adaptation of the phenotype—with ecology because it focuses on phenotypic attributes that confer fitness differences across environmental space (see Trait Combinations and Interactions, below); these are the outcome of both natural selection and stochastic processes that can explain species distributions, occurrence or abundance, and community structure.
- Third, it is scalable, since traits can be linked to acclimation if they are labile, explaining community composition and richness with respect to intraspecific trait variation, or explaining interspecific compositional turnover where environmental change (spatial or temporal) causes the declining and increasing fitness of species at the boundaries of their trait differences (see Spatial-Temporal Scales, below).

As well as bringing these points together, trait-based approaches may also offer a route through problematic application of lichen systematics to lichen ecology, in which taxonomic concepts may become difficult to apply in practice, as exemplified by a lack of discernible field characters among known species [19,20,21]. A focus on traits may thus avoid lichens becoming an increasingly narrow specialism within ecology, offering instead a practical route towards integration if a spectrum of functional traits can be identified and applied by non-specialist ecologists. Consequently, trait-based approaches are arguably more powerful, despite being more user-friendly in application [22]. Similarly, while ecological conclusions may be difficult to generalise if drawn from the analysis of species names, given differences in regional species pools that reflect independent evolutionary or biogeographic histories, the use of traits provides opportunity for recognising general ecological rules that govern the way in which communities are assembled, despite species pool differences.

To date, the application of trait-based research to understanding lichen distributions, occurrence or abundances, and/or lichen community structure, has been demonstrably effective and this is highlighted through our selection of examples below. Nevertheless, the research groups that have been using trait-based approaches lack a common methodological approach; contrasts include the nomenclature applied to classification of different traits, through to differences in the observed relationship of traits to environment response and ecosystem function. There is still much to discover. Although certain traits are now embedded and routinely used in explaining lichen response, such as the physiological difference between green-algal chlorolichens and cyanolichens [23,24], recent studies also show that, within these categories, there are relevant physiological and functional differences that can more finely resolve the lichen response [25]. There is emerging synthesis around other potentially generalisable traits, especially the lichen response explained by links between phenotype and thallus hydration [26], although again key challenges often remain, including the way in which structurally different phenotypes can converge on the same hydrological response [27]. There are methodological questions to be solved about how this convergence in response is recognised and dealt with analytically.

Recognising both the significant opportunity provided by functional trait studies, and the challenges involved, and without wanting to provide overly prescriptive answers, this paper provides a review of trait-based research in lichen ecology, pointing to a series of recommendations that can move the field forwards. It is drawn from the experience of multiple authors, particularly with respect to European ecosystems from the Mediterranean to the Arctic-Alpine. It emphasises the types of traits that lichenologists have used in their studies, both response and effect, the bundling of traits towards life-history strategies, and the critical importance of scale (both spatial and temporal) in functional trait ecology.

## 2. Types of Traits

Functional traits can be considered in two ways. First, they are the phenotypic attributes that explain the ecological response of a species to one or more biotic or abiotic, spatial or temporal gradients constituting the environment. As stated above, these response traits [28] will control fitness differences across environmental gradients (rates of establishment, survival, reproduction), thus affecting lichen species distributions, occurrence or abundances, and cumulatively across species, community structure. If well characterised, these traits can subsequently provide proxy information on their environmental controls, being useful to monitor environmental change in space and time, including changes that may be more difficult to study by other means (such as for air pollution).

**Figure 1:**
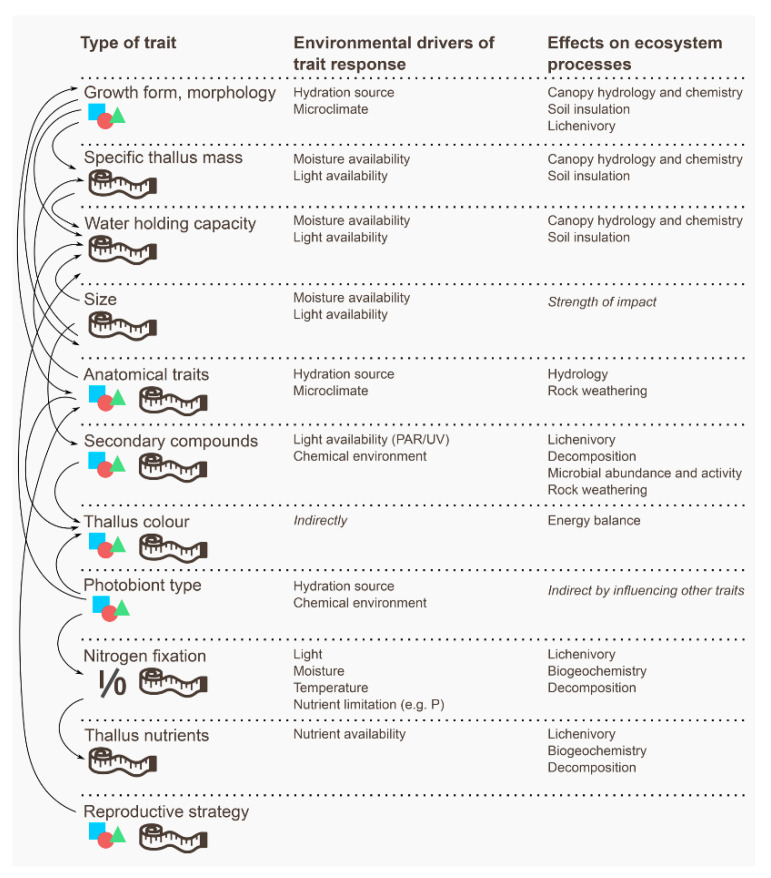
Schematic to show contrasting types of traits that might yield qualitative (shapes) or quantitative (measuring tape) data, and their potential relationship to environmental drivers controlling a species/community response, and/or the effect on ecosystem processes. Arrows show an a priori understanding of linkages between traits (e.g., photobiont type also affects water holding capacity). Note that thallus colour is indirectly related to the environment through associated traits such as photobiont type or secondary compounds. Additionally, that reproductive strategy associates with lichen anatomical traits but is separated out because of its link through to demographic processes that also affect community composition.

Second, functional traits may describe the effect of a species on ecosystem processes. These effect traits [28] are focused on how a shift in lichen species distributions, occurrence or abundances, and/or community structure, can have consequences for ecosystem functioning, such as lichen nitrogen fixation relevant to terrestrial biogeochemistry, through contribution to nutrient cycling [29,30,31]. These response and effect categories are not mutually exclusive (Figure 1), and some traits reflect both response and effect simultaneously; accordingly, response and effect traits may, but do not necessarily, overlap.

### 2.1. Response Traits

In principle, the ‘trait space’ that lichens might occupy is highly dimensional, since it can combine a wide array of different phenotypic attributes that are measured to varying degrees of precision [32,33]. Key foci in the search for lichen functional traits have included gross thallus morphology and anatomy, the identity of photobionts, attributes linked to hydration and physiology such as surface hydrophobicity or water holding capacity, reproductive mode, and thallus chemistry as nutrient status, secondary metabolites or tissue pH, etc. [32,33]. The search for traits has been related to natural constraints such as drought [34], or anthropogenic drivers such as air pollution [35,36]. The terminology used to organise and think about traits (Table 1) has encompassed the difference between traits that are easier or harder to measure (soft versus hard traits), those drawn from primary data or bibliographic, whether they are quantitative or qualitative, whether they are directly related to physiological response, being proximal, or proxies for response, being distal or compound traits. In providing our introduction to response traits, we here explore some of the different ways in which lichen traits have been organised into the ecologist’s toolkit.

#### 2.1.1. Explanatory Power or Information Assembly Effort

Trait-based studies often need to strike a balance between the effort required to measure a given trait and its explanatory power. This links to the flexible but subjective concept of ‘hard’ and ‘soft’ traits [32], which are defined by the expenditure (amount of time or money invested, or expertise required) to measure and apply a given trait within a study. In this regard it is important to recognise an ambiguity that brings into question the usefulness of these terms; the measurement of certain traits such as water holding capacity could be relatively easy and not especially time consuming when applied to a foliose lichen (soft trait), while it may, in practice, constitute a hard trait when measured for squamulose species intermingled with soil. There is also a potential confusion of terminology. In the wider plant literature [28] hard traits are considered those which capture a precise function (e.g., nitrogen fixation or litter decomposability), and it is important to be aware of the way in which trait terminology may have been differently applied across ecological domains.

Furthermore, the higher cost involved in measuring certain traits may not always contribute additional information within a hypothesis, compared to insights gained from using more easily obtained trait data. Thus, Hurtado et al. [37,38,39] found that readily applied categorical traits of epiphytic lichens, such as the growth form and type of photobiont (considered at a high level of systematic classification), were highly relevant in explaining lichen response to macroclimate across a latitudinal gradient in Europe. Surprisingly, traits with a more direct physiological association (e.g., chlorophyll a, water holding capacity, specific thallus mass, tissue %N or %C) were little or not relevant at this scale of analysis. These harder to measure traits may nevertheless be related to smaller-scale environmental variation, with a response signal that is obscured by coarse-grained macroclimatic data.

#### 2.1.2. Qualitative versus Quantitative Traits

Almost all traits can be assessed either as quantitative or qualitative. For example, metabolite profiles could be considered in the form of a presence/absence matrix (relatively low cost), or concentration ranges for each metabolite could be measured and used in a quantitative form (relatively high cost). The use of presence/absence data for metabolite profiles in lichen communities is common practice (e.g., [22,40,41]), but the relative benefits of switching to quantitative metabolite data, with respect to the explanatory power of results, remains to be tested. Similarly, growth forms of lichens represent categories within a continuum. Using a qualitative approach requires an a priori decision over the scale at which differences in growth form are recognised. This is often unacknowledged (such as when growth forms are bibliographic, and drawn from flora accounts), while the number of categories may also differ substantially between studies (e.g., foliose, against foliose broad or narrowly lobed). Quantitative measures reflecting morphological length/width relationships may provide more generalisable data [42], though can be challenging to integrate across species that have a fundamentally different growth form (e.g., fruticose versus crustose species). Studies can often be recognised as aligning themselves along a gradient of trait categorisation that is either more or less finely resolved (cf. [43,44,45]).

#### 2.1.3. Bibliographic or Primary Data

Quantitative traits are often based on primary observations made for a particular lichen population or study area but for qualitative traits it can be common practice to combine field knowledge with information from bibliographic sources, such as original research papers, regional floras (e.g., [46,47]), or databases and data portals, e.g., LIASlight [48], CNALH [49] and ITALIC 6.0 [50]. However, there are often important differences in detail; metabolite profiles in floras and databases are generally coded as presence/absence or in a semiquantitative way (such as describing compounds as trace or accessories versus the main component), but with detailed concentration ranges absent. With the exception of LIASlight, none of the aforementioned resources aims at providing a global representation of lichen traits. This can cause problems because even seemingly simple traits, such as growth form, can be ambiguous when studied over large geographic scales; the prevalence of different types of photosymbiodemes for the same lichen-fungus is the extreme example of this change under different environmental conditions. Reproductive mode (presence of fruiting bodies, in addition or instead of various forms of vegetative propagules) is likewise easily accessible via bibliographic sources, though can also vary substantially within a species across regions. Borrowing from niche theory, one might contrast the fundamental trait (the potential biological range of an attribute), with the realised trait needing to be validated by observations within the study area of interest.

#### 2.1.4. Proximal or Distal Traits

Again, borrowing from niche theory [51,52], traits may be considered relatively more proximal if they tend towards a direct link with the lichen physiological response, as may be the case for specific thallus mass [53,54]. Or traits may be distal, if they capture response or effects more broadly, such as using growth form. In the latter case, traits may be compound if they summarise responses across different aspects of lichen biology related to multiple environmental variables (see Trait Combinations and Interactions, below). As an analogy, the light response curve of lichens or plants is a very direct physiological response, intermediate might be the specific thallus mass, or for plants the specific leaf area, while compound may be the growth form (fruticose, foliose or crustose), or for plants similar categories such as Raunkiær’s life forms (phanerophytes, chamaephytes, hemicryptophytes, etc.). Partitioning the particular relevance of distal/compound traits to any observed trend can be challenging; for example, the lichen growth form may relate to water use strategy and physiology (cf. [42,53,54]), though may also have implications for successional processes through competitive species interactions [55], while reproductive mode such as the presence of isidia might affect spatial-temporal response to habitat availability through dispersal limitation [16,56] as well as affecting physiology through thallus gas exchange [57,58].

#### 2.1.5. Variation among Individuals

Advances in lichen trait studies are exploring the importance of functional trait variability among individuals within a population, as well as among species. The range—or plasticity—of a given trait has consequences for acclimation that may link importantly to the species niche [59,60]. For example, since lichens are compound organisms, the specificity of the fungal-photobiont association, and an exploration of this range as a functional trait itself, can reveal acclimation potential through photobiont selectivity to local environmental conditions, creating resilience to environmental change [61,62]. The ecological potential for selectivity is determined by differences in the evolutionary specificity of the lichen fungus, varying from strict specialists to broad generalists (e.g., [63,64,65]). Generalist species may have advantages across different environments, with more specialist species having narrower geographical distributions and ecological niches [63,64,65].

The study of lichenometry provides a salient warning about variability in traits below the species level, with respect to growth rates and size. Growth rates and maximum thallus size have long been used in relation to and as a proxy for the time span since major physical disturbance [66,67]. This rests on the assumption that regional calibration of growth rates, within a comparable environment, is possible through analysis of thallus size patterns on substrata with known history. Some knowledge of variability in growth rates and thallus size is important to confirm this assumption. However, new data on cryptic speciation, even with taxa popularly used for lichenometry, such as the *Rhizocarpon geographicum* aggregate [68], calls into question the standardisation by targeting at a putative species level. Instead, selecting candidate thalli for lichenometry based on homogeneity in relevant traits (growth rates and maximum thallus size) may offer a way out of this problem and in fact may be—albeit unconsciously—already in practice when using morphology-based concepts for taxa such as *Rhizocarpon geographicum* auct. (e.g., [69]). Furthermore, standardisation of growth rates and thallus size requires that multiple covariables, such as the effects of reproductive stage, substratum type and variation in microclimate also be taken into account [70,71]. Having achieved this sufficient level of control, observation of small-scale temperature effects on the freshwater lichen *Dermatocarpon rivulorum*, in a closely monitored stretch of subalpine watershed, has led Shivarov et al. [72] to propose variability of thallus size and colony extension as a trait for monitoring the effects of climate change.

In summary, recent studies from both a microhabitat scale [73] to biogeographic scale [37,74] have shown that incorporating trait variation for individuals, below the species level (≈intraspecific variation), is important when explaining the response of lichen communities not only to microclimates, but also to macroclimates across wide geographic extent. Thus, Hurtado et al. [37], with respect to community structure, and contrary to the general pattern expected for plants, showed that intraspecific variability in functional traits explained the community pattern of epiphytic lichens along a 3000 km latitudinal gradient in Europe, being a larger effect than species turnover. Intraspecific variability should be taken into account in order to fully understand the effect of environmental change on lichen communities.

### 2.2. Effect Traits

Most trait-based lichen studies have explored response traits; there is a major knowledge gap in our understanding of how these traits may also link to ecosystem function and the delivery of ecosystem services. This requires investigation of the role of effect traits. In many cases response traits may coincide with effect traits, and changes in the trait composition of a community, in response to environmental factors, can ultimately result in a change to how the lichens affect ecosystem function. For this same reason, Lavorel & Garnier [28] proposed a conceptual framework that linked the response and effect traits of plants. They suggested that response traits related to resource availability may be congruent with a direct effect on nutrient cycling, for example. In contrast, traits linked to disturbance, and reflecting demographics, have generally a weaker relationship to ecosystem processes. Whether this framework is broadly applicable to lichens has not been thoroughly tested, despite the fact that lichens play important roles in driving ecosystem function [75] and in supply of ecosystem services [76]. With respect to effect traits, it is noteworthy that conservation policy is increasingly focused away from biodiversity per se, and towards the link between biodiversity and ecosystem services and goods. An improved understanding of lichen effect traits is likely to become a priority therefore, to better understand the role lichens play in ecosystem functioning, and to secure the ecosystem services they provide. This would incorporate the benefits to humans that are derived from lichens, which, when referenced below, are organised using CICES, the Common International Classification of Ecosystem Services [77].

**Figure 2:**
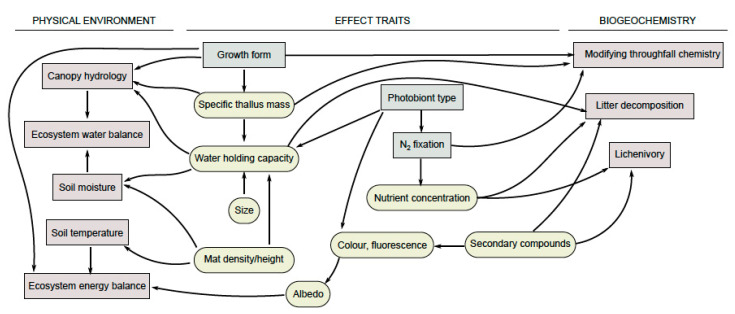
A schematic to suggest how lichen response traits, especially those linked to the key physiological parameter of thallus hydrology (growth form, photobiont type), can also affect the physical environment of energy and water balance as a regulating ecosystem service, while also linking to biogeochemistry as a supporting ecosystem service. As regards the effect traits, boxes represent what are routinely categorical and ellipses continuous measurements.

As hypotheses to provoke future research (Figure 2), we here offer the proposal that response traits determining how lichens respond to water availability (e.g., water acquisition and water storage) are also effect traits for how lichens influence the physical properties of the ecosystem, i.e., acting as a regulating service in the water cycle, while also altering the energy balance and microclimate. This represents a regulating ecosystem service. We also suggest that traits determining the lichen response to nutrient availability are important for how lichens influence biogeochemistry. This represents a supporting ecosystem service. As an additional consideration, traits related to growth form and visual aspect may also be critical in the provision of cultural ecosystem services.

#### 2.2.1. Regulating: Water Availability and Energy Balance

Ground-dwelling (terricolous) lichens directly influence soil microclimates through their physical properties. This influence is strongly linked to thallus morphology, though questions of scale become important (see Spatial-Temporal Scales, below). For example, traits measured at the scale of a population or community, such as ‘mat-level’ traits of terricolous species, are probably more relevant to regulating services than the variability of individual thalli. For instance, lichen mat density and mat water holding capacity are negatively correlated with soil temperature [78,79]. Nystuen et al. [80] also found lower soil temperature under lichens with greater mat thickness, though with these population- or community-level effects unrelated to individually measured attributes. Studies on bryophytes also show that heat transfer through mats is regulated by mat thickness and moisture content [81]. Further, where lichen mats affect the radiation budget by changing albedo, lichen colour (and metabolite profiles, see Types of Traits, above) becomes important, affecting surface temperature [82,83]. Aartsma et al. [84] confirmed that species composition in lichen-rich heath influences albedo and microclimate at the habitat scale.

Morphological attributes can strongly affect thallus water uptake and release, again scaling at the population- or community-level to regulation of the water cycle [85]. Several studies have shown that epiphytic lichens can affect canopy hydrological parameters (see [86]). As such, Pypker et al. [87] showed that epiphytes store 3-5 mm equivalent of rain in an old-growth Douglas-fir forest. Lichens may also reduce stemflow and throughfall, but studies on this remain inconclusive [86]. In fog-driven ecosystems, epiphytic lichens may improve host plant water use through increased fog interception [88]. Most studies regarding the lichen influence on canopy hydrology have not distinguished between species however, making it difficult to directly link these larger-scale processes to individual lichen response traits. Nevertheless, it could be assumed that the key morphological traits related to water uptake and retention [26] are important as effect traits in this context.

Given the evidence to date, it seems a reasonable proposition that the energy balance and ecosystem water economy of lichen dominated habitats emerges from the aggregation and up-scaling of traits that originate with the morphology or structural complexity of individual lichen thalli. Conceptually, lichen morphological studies are now integrating at least two tightly linked continuous response traits, relating strongly to water uptake and release kinetics at a thallus-scale [26,54]—specific thallus mass and water holding capacity—but which are pivotal for how lichens might affect ecosystem hydrology and insulation. As morphology, specific thallus mass and water holding capacity also respond to environmental gradients, e.g., water and light availability [89,90], this is an example of the potentially strong connection between response and effect traits.

#### 2.2.2. Supporting: Biogeochemistry

In addition to the effect on hydrology discussed above, lichen epiphytes may alter throughfall and stemflow chemistry and thus play an important role in nutrient cycling [91,92]. More broadly, lichens can significantly affect biogeochemical cycles through surface weathering, nitrogen fixation and by trapping minerals from dry and wet deposition [92,93]. The rates at which these processes occur is possibly influenced by lichen traits, although our knowledge of these processes is limited. For instance, lichen rock weathering is certainly influenced by an amalgam of traits [94,95]. Growth form may affect rock weathering, and crustose lichens (especially endolithic examples) often have greater hyphal penetration into rocks than other forms. However, foliose and squamulose species, and especially those with rhizines, may also contribute significantly to rock weathering. Further, thallus expansion and contraction are greatest in gelatinous lichens. Dark-coloured lichens may induce rock weathering by increasing microthermal gradients and thus the number of freeze–thaw cycles [96]. Both qualitative and quantitative variation in metabolites also influence weathering [94]. Ultimately, these trait-determined effects will play a critical role in soil formation and quality, cumulatively over long timescales. The contribution to soil formation may be facilitated by the nitrogen fixation of lichens with a cyanobacterial symbiont [29,30,31]. As a contrast, the lichen weathering of historic monuments has been viewed as a disbenefit, although some dispute exists as to whether lichen colonisation is offset by thermal insulation and impermeability, thus providing protection of stone [97].

Carbon and nutrients fixed by lichens are ultimately released to the ecosystem through litter decomposition or consumption by various animal groups. There has been a growing interest in how lichen traits drive the rate of these processes (as reviewed by Asplund & Wardle and van Zuijlen et al. [75,98]). Lichen palatability is governed by qualitative and quantitative variation in metabolites [33,99,100,101]. Although thallus nutrient concentration seems to have little effect on grazing by molluscs, for example [33], it plays a role in regulating the abundance of other invertebrates in lichens [45], with potential consequences across food-webs [102]. Geiser et al. [103] developed a model for estimating the loss of forage lichens (e.g., with a role on critical winter forage for ungulates) following nitrogen deposition in US forests, identifying levels of risk to be taken into consideration in land management.

As final considerations, the reduction of cover and diversity in lichen-dominated biocrusts is shown to lower the capacity to sequester atmospheric CO_2_ [104] while lichens can affect successional processes including the establishment and performance of vascular plants [80,105], and growth form also influences lichen ability to prevent erosion, by retaining soil particles [106,107].

## 3. Trait Combinations and Interactions

Although studies to explain lichen distribution or community structure often consider more than one type of functional trait, many such studies treat these traits as separate individual explanatory effects. This additive approach may be entirely appropriate if the study is focused on a single (or few) strong gradient(s), but there are potential advantages to ‘bundling traits’, considering their combined and interactive effect, to achieve an integrated evolutionary and ecological perspective. Here, we explore the benefits of bundling traits, highlighting the advantages of this approach, and the challenges of why and how to do it.

### 3.1. Why Bundle Traits?

By bundling traits, we mean considering more explicitly how separately defined functional traits (e.g., specific thallus mass, metabolites, reproductive mode) are interrelated through their evolutionary history, and interact at ecological scales. Lichen species are structurally complex and while the prior cause of a lichen phenotype can be difficult to explain, evolution will nevertheless have operated on the whole phenotype, as natural selection shaped the adaptive capacity of individuals towards effective establishment, survival and reproduction. Individual traits have co-dependent evolutionary histories and shared ecological benefits or constraints, that together determine the lichen response to the environment, and explain lichen distributions, occurrence, abundances and/or community structure. The integration of functional traits, in determining the lichen response to environment, can therefore reflect key linkages between evolutionary history and ecological process, potentially increasing the explanatory power of trait analysis.

### 3.2. An Evolutionary Niche Model

Of critical importance in motivating the analysis of combined and interacting traits is the way in which this trait bundling keeps our studies consistent with models of the evolutionary process. The classic habitat template model of the niche [108,109] describes a process in which the habitat—the amalgam of biotic and abiotic environmental conditions—is a template into which evolution by natural selection shapes the organism’s phenotype through adaptation that maximises fitness (establishment, survival, reproduction). This region of fitness is the species niche. Here, in a logic that is internally consistent, the niche links the environment to the evolutionary process of adaptation, explaining the consequent phenotype, and therefore provides a phenotypic explanation of species response to the environment (as measured by ecologists, using functional response traits). This model is consistent with and supported by evidence of convergent evolution, in which unrelated species evolve/acquire similar phenotypes, as they adapt independently to equivalent environments. In a very broad sense, the moisture-harvesting morphology of certain pendulous lichens such as *Usnea longissima* or *U. dasopoga* appears remarkably similar to that of unrelated taxa such as *Tillandsia usneoides* (Spanish Moss), while the water-retaining hypothallus of lichens such as *Pectenia* spp. [110] is similar to the root velamen radicum of epiphytic orchids [111,112]; these may be cases of homoplasious adaption to the water-limited epiphytic habitat. However, among unrelated lichen-fungi there has also been striking convergent evolution [113] including in morphology and reproductive structures [114,115,116]. Furthermore, because we are dealing with the evolution of a composite organism, shared photobiont specificity might also be considered as a functional trait that is potentially convergent across unrelated taxa [117], with these symbiotic relationships recently being extended to consideration of the entire microbiome [118].

How might the habitat template model benefit an understand of lichen distributions or community structure? Since the habitat presents the complexity of environmental conditions into which the whole phenotype has evolved and adapted, one should expect multiple functional response traits to have coevolved (and co-occur) in a way that reflects their combined adaptive value. In other words, the model of an ‘environmental filter’ does not operate as effectively on single traits, but rather on organisms that are constructed of multiple traits, the combination of which dictate species performance. This explanation becomes increasingly important as the environmental parameters investigated by the ecologist become multivariate, and the functional traits required to understand responses become multidimensional. For example, there are broadly different occurrence/abundance patterns for cyanolichens and chlorolichens in relation to the distribution of moisture [119,120,121], explained by the widely cited difference in the cyanolichen requirement for liquid water in photosynthesis, compared to an ability to recover photosynthetic activity through uptake of water vapour for chlorolichens [23,24,122]. However, a spatial environmental gradient in moisture is likely to covary with the light regime; open dry habitats compared to shady moist habitats. Consequently—alongside and potentially interacting with the photobiont—there will be relevant differences in lichen species response to photosynthetically active photon flux density (PPFD) [123] or colour pigmented photoprotective mechanisms [124] that start to form a bundled set of traits. Extending the role of the phenotype further, and beyond the physical environment (moisture or light), there are biotic interactions also to be considered. Lichens with cyanobacteria tend to have relatively high specific thallus mass (STM), and higher water holding capacity per unit STM [42,90], which facilitates time spent hydrated and physiologically active, but which may also link to biological interactions and their competitive ability in overtopping competitors for space [55]. Furthermore, the occurrence or abundance of lichens contributing to a chronosequence of community structure can be partly explained by a species’ reproductive mode and dispersal regime [125,126], while reproductive mode facilitating early or late colonisation may associate with an investment in longevity such as through the synthesis of metabolites [127]. As this consideration of bundled traits starts to become richer and potentially more complex, life-history strategies emerge as a means of summarising multivariate functional response traits through reference to evolutionary trade-offs.

### 3.3. Life-History Strategies

Overall, the bundling of traits described above, as relevant to a spatial-temporal response, resonates to some degree with the *r-K* concept of life-history strategies [128,129], describing a trade-off between short generation times and rapid colonisation, and longer-generation times and competitive ability. Similarly, an apparently parallel response to gradients of light/moisture and the pollution regime [130,131], could be viewed as partially analogous to the stress-gradient derived from the C-S-R model of life-history strategies [132,133]. It is tempting therefore to reduce the high-dimensional space occupied by combinations of interacting traits into fewer life-history strategies that reflect key trade-offs in environmental adaptation (cf. [108,109,129,132]). However, this poses some additional challenge. First, the primary models of life-history strategy have been developed with taxonomic bias towards, for example, animals and plants, and their relevance to a group with as complex an evolutionary history as lichens [134], remains to be fully tested (though see, for example: [135]). Second, the life-history strategy approach is so general that it is arguably too blunt a tool, and the flexible search for specific trait combinations that are relevant to a particular question, allowing for a specific context of environmental gradients and scale, may be more insightful (cf. [36,136]). This flexibility poses its own challenges, however.

### 3.4. Conceptual Advances and Challenges

Consistent with our argument that functional response traits may be strongly linked through their shared evolutionary history, methods in trait analysis also clearly advocate that this non-independence of traits, shared among phylogenetically related species, needs to be accounted for (though see [137]). For example:

- One needs to take account of phylogenetic relationships, based on the assumption that closely related species share underlying similarities (they are not evolutionarily independent), with this non-independence incorporated into tests of trait-environment relationships to avoid statistical error, i.e., erroneously high degrees of freedom, type I error [138];
- One might also include phylogenetic relationships in order to account for similarities among species that exist because of niche conservatism, but that are unmeasured by the traits being used; effectively using phylogenetic clustering as a proxy for unmeasured functional traits, and prompting a search for new functional traits [139];
- One might further include phylogenetic relationships to strengthen understanding of community assembly, as over- and under-dispersed phylogenetic clustering [140,141], though interpreted in the context of macroevolutionary processes such as convergent evolution or adaptive radiation.

This integration of phylogeny, traits and community structure can now be implemented by various methods, e.g., phylogenetic generalised least-squares linear models (PGLS) and phylogenetic generalised linear mixed models (PGLMM) [142,143], and these have been applied within recent lichen studies that innovatively integrate phylogeny and trait analyses [38,144].

An additional challenge is posed by traits that are dissimilar in appearance, but which are functionally similar, considering the difficulty of recognising and accommodating these, such as the role of (pseudo)cyphellae (e.g., *Pseudocyphellaria, Sticta*) or pores (e.g., *Melanohalea*) in thallus hydration and gas exchange. This could be extended to traits that have several, but quite contrasting functions, such as the relevance of diaspores (isidia/soredia) to both dispersal and gas exchange [57,58]. Furthermore, it is possible that traits interact in such a way that the role and relevance of a trait could shift depending on the environmental context. A reasonable example could be the preferential occurrence of cyanolichens in relatively drier environments (skewed away from their moisture niche optima), if, for example, these have lower throughfall nitrogen loading than wetter environments, from which cyanolichens are excluded by a physiological sensitivity to air pollution.

## 4. Spatial-Temporal Scales

A lesson emerging from our review is that the initial trait choice for ecological investigation needs to consciously address the scale of biological organisation, such as the trait variability within a single individual, variability among populations within a species, among species and among communities. Depending on the research question, traits will typically be assessed within a certain part of this spectrum, by carefully matching the scale of biological organisation with the environmental scale of interest. This matching is one of the key challenges of trait analysis [145] since traits may respond to environmental drivers across different scales. For example, climate variables might influence lichen species through a functional trait response that has signatures at a regional scale, while the same signatures may also be found at a nested microhabitat scale, as species respond to locally suitable microclimates that can be outliers within a sub-optimal climatic regime, as has been shown for cyanolichens and cyanolichen communities [146,147].

**Figure 3:**
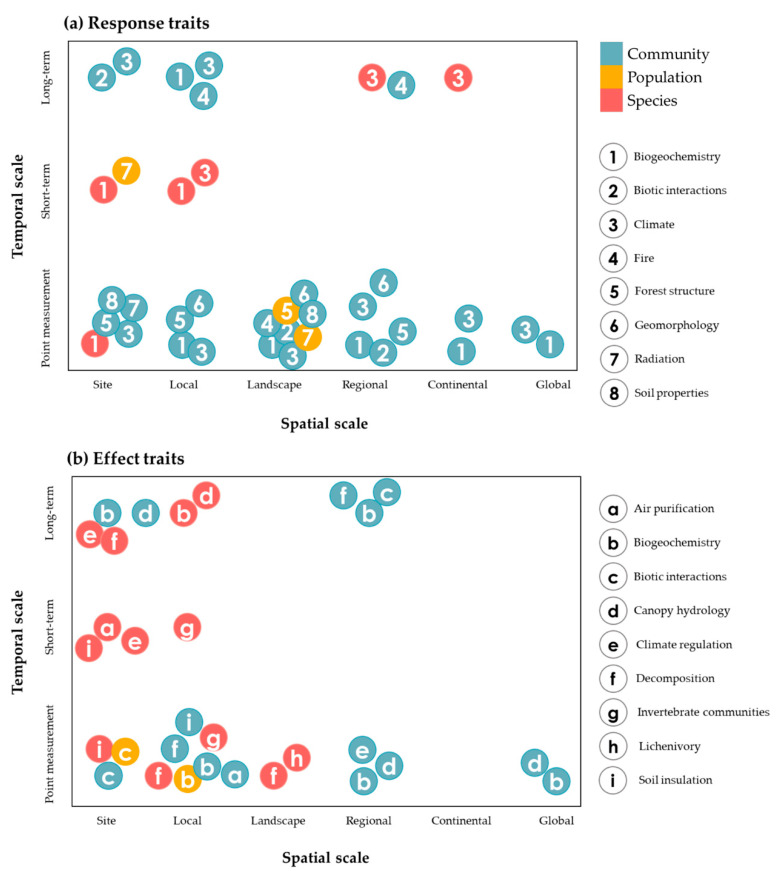
A sample of trait-based lichen studies, comparing the use of temporal and spatial scale (vertical and horizontal axes), when considering the scale of biological organisation (colour) and the environmental factor(s) of interest (numbered) for (**a**) response traits and (**b**) effect traits. The plot is supported by the review of studies presented in Supplementary Tables S1 and S2.

Research design must therefore consider how the environment might influence across scales to affect lichen distributions, while also accounting for scales of biological organisation that inform the trait analysis. As a starting point, one might ask, what are the environmental factors that, for a given spatial and temporal context (being scale-dependent), are limiting the performance of lichens, their establishment, survival, reproduction, as revealed through the concurrent analysis of traits appropriate to that scale? We reviewed the recent literature to understand how authors might have approached temporal and spatial scale, while also considering the scale of biological organisation and the environmental factor of interest (Figure 3). Although not exhaustive, it is apparent that the greatest number of studies for both response traits (Figure 3a) and effect traits (Figure 3b) have been for individual time-points applied across all spatial scales (from local to global), irrespective of environmental factor; and while community-scale has been the dominant focus for response traits, this is balanced by a similar number of species-scale studies when effect traits are considered.

We explore the issue of scale further, by discussing specific examples below.

### 4.1. Biological Scale

The biological scale can be expanded into a hierarchical framework in which structures are defined by part-whole relationships. Entities at higher levels are composed of parts from lower levels. In this classical system, levels of biological organisation include the atomic, molecular, cellular, tissue, organ, organismal, group, population, community, ecosystem, landscape, and biosphere; components that are structured hierarchically in space and time. Higher level abiotic conditions, such as macroclimatic variability and regional biogeochemistry, can impose constraints on lower-level biological processes [148]. However, following the principle of emergent properties, each successive level of biological organisation can acquire attributes that are not simply the sum of observations made in a previous lower level [149]; consequently, it is important to choose traits at a level of biological organisation that reflect responsiveness to the appropriate environmental scale. This choice is not always easy. For example, in understanding the desiccation curves for a single thallus, its anatomy, morphology, and particularly upper surface characteristics such as the presence of a cortex are important [150]. However, when multiple thalli of the same species coexist as a population, then shared morphological complexity can lead to an extended phenotype [151], pointing to the need for a more aggregated, higher-level analysis of traits. As a caveat, other poikilohydric organisms, such as some aquatic bryophytes, including *Fontinalis antipyretica*, have been wrongly classified as desiccation intolerant based on experiments applied to single shoots [152]. Although the level of relative humidity at which these experiments were performed was realistic, the rate of water loss was unrepresentative of natural conditions, because tests were performed on isolated leaves or shoots. In contrast, under field conditions, this aquatic moss is known to survive summer periods of Mediterranean drought (2–3 months) when it stands out of water owing to decreased river flow [153]. Consequently, in performing the same desiccation experiment though using the entire moss colony, instead of single shoots, the outcome was different, and the moss was classified as being desiccation tolerant in line with field knowledge. Simple features of bryophyte morphology and life form, instead of individual leaf traits, can more effectively capture the ecological adaptation of bryophytes to habitats with different water availability.

Although certain levels of biological organisation may be more relevant to answering a specific research question than others, it is important not to overlook the potential for trait variability at one level of biological organisation to affect responses at different scales of analysis. A study assessing variation of traits related to photosynthetic performance, nutrient cycling and water use in epiphytic lichens across Europe showed that interspecific variation (order and species level contrasts) explained a proportion of lichen response overall [38]. However, when trait variation was analysed at the level of community structure, intraspecific trait variability made a higher contribution than interspecific differences even at this continental scale [37]. In helping to guide researchers through the issue of scale, and with respect to the impact of anthropogenic drivers (such as air pollution and climate change), Branquinho et al. [154] proposed different biological scale measures depending on the intensity of the driver: (i) Ecophysiology-based metrics for low intensity drivers affecting organisms’ individual performance; (ii) Trait-based metrics being reserved for medium intensity drivers that differentiate the ecological performance of sensitive compared to tolerant species, causing shifted abundances and resulting in community-level functional properties; and (iii) Taxonomic-based metrics for broader scale impacts which may culminate in species loss [154]. It was proposed that impacts on lichens should be measured, from the highest to the lowest levels of biological organisation, focusing on the initial level of organisation that shows significant variance.

### 4.2. Spatial Scale

It is axiomatic that spatial scale plays a central role in ecological questions and conclusions [148]. The two main characteristics of spatial scale are: (i) Grain, defined as the minimum spatial resolution of the data and (ii) Extent, defined as the scope or domain of the data. Spatial scale is a central consideration since it forms a perspective that governs both the observed pattern of diversity and its dominant ecological processes. Especially considering the potential longevity of lichens, which ties them to deep temporal scales (across decades or centuries), their distributions, occurrence or abundances, and community structure, may be the outcome of multiple environmental drivers operating across multiple scales. Since lichens have been widely used as environmental bioindicators, lessons relating to spatial scale can be drawn from the rich canon of work addressing global change.

Traits have been investigated with respect to the larger-scale (macroscale) environment (cf. [39,155,156]; and for ecosystem functioning see [85,93,157]); for example, in relation to climate sensitivity, Matos et al. [34] suggested that photobiont type was particularly sensitive to aridity, highlighting its role in understanding the lichen response to and their potential as indicators of climate change. This macroscale sets the scene for nested and regional effects (mesoscale) such as land-cover classes that might explain differences among lichen functional groups, inferred from their ecological preferences for nutrient availability [158]. Thus, the distance over which nutrient regimes might influence lichen functional groups can depend on land-cover type: (i) ‘Annual cultures’ had their maximum influence at distances of 600 m; (ii) ‘Bare-lands’ at 3400 m; (iii) ‘Artificial areas’ at 1800 m; (iv) ‘Ocean’, which may account for factors such as salt spray, altitude and humidity, had the largest scale of influence over lichens, ranging from distances of 1800 m to more than 6600 m [158]. This nesting of effects within a given broader scale again highlights the importance of considering how lichen response is affected by different environmental variables across scales. To illustrate this point further, at a similar mesoscale, Prieto et al. [144], in an analysis of beech forests in the Iberian Peninsula, found that environmental filtering and local species interactions regulated lichen communities differently under contrasting environmental conditions. Likewise, Hurtado et al. [159] found contrasting factors controlling the functional composition of lichen communities measured at the local scale, for different biogeographic regions, again suggesting that environmental drivers have a cross-scale spatial interaction. These studies also remind us that biodiversity is the outcome of processes operating across locales (across their granularity, for a given extent). Beta diversity patterns for Lobarion communities in Italian forests were significantly determined by factors relating to the forest structure [160], but with the relative importance of the different structural features dependent on the spatial scale of observation.

The microscale is represented by its amalgam of local chemical-physical characteristics such as might be encountered on a tree, or on stonework, representing the fundamental habitat for an epiphytic or an epilithic lichen, respectively. At this smaller scale, several works have reached beyond occurrence/abundance patterns, to investigate demographics, including population structure and species fecundity [161,162]. For example, Rubio-Salcedo et al. [163] showed that tree species was the most important factor in generating different patterns of establishment, revealed through intraspecific variability in traits such as thallus size and reproductive capacity for *Lobaria pulmonaria.* However, as with nested effects at the regional scale, interactions can play a fundamental role at the microscale, as demonstrated for a study analysing the structure of *Lobaria pulmonaria* populations in different forest types in Italy [164]. The tree-level probability of occurrence was influenced by the interplay between the forest habitat type and abiotic and biotic factors whose interactive effects varied during the life cycle of the lichen, again revealed through intraspecific trait variability such as for thallus size. Thus, the effect of forest habitat type was significant only for adult thalli while the early life stages were habitat-independent and were strictly associated with tree-level factors. While experimental tests have studied the relationships between water availability and the eco-physiological response of lichens, with size (≈life stage) as a key trait [110,161], knowledge of the role of intraspecific traits and interactions at the microscale is generally limited (though see [73] for insightful analysis). This is mainly due to the difficulty of assessing, in a cost-effective way, the microhabitat features that characterise the microenvironment. In a few studies, microenvironment is quantified; Giordani et al. [156] modelled the occurrence of epilithic lichens in response to light and water availability, to reveal a clear alternation between foliose and fruticose, and crustose growth forms, with decreasing light, suggesting that an excessive availability of water reduced the presence of foliose lichens.

One of the ecologist’s key methods for understanding spatial processes is to use the landscape as a natural experiment, and to focus on situations where environmental gradients can be isolated. In a study on north and south slopes of limited topography (300 m altitude), lichen traits related to water requirements were patterned into contrasting microclimatic conditions (as controlled by topographic contrasts) despite the close spatial distance between sites [165]. On northern slopes, lichen traits reflected higher water availability, explaining a 13% shift in community composition when compared to southern slopes [165]. There are situations where insights from carefully chosen study systems have been translated to larger scales. The effect of atmospheric pollution on lichens, leading to widely applied critical loads for nitrogen deposition, have been based on local trends in lichen community composition [166,167]. Aimed at integrating different functional groups, different scales, and different temporal ranges, Bowker et al. [168] proposed a multi-scale, hierarchical conceptual model that could predict lichen–moss occurrence and composition as a function of climate and soil variables at five spatial scales on the Colorado Plateau.

### 4.3. Temporal Scales

Temporal scale (with its own resolution of grain and extent) may refer not only to the pace of environmental change, but also to the time lag between the driver and the response. In this way, different aspects of the temporal context can appear to be more or less important in explaining lichen distributions, occurrence or abundances, and community structure. The detection of temporal variation may be one of the most demanding considerations in ecological research due to the difficulty in compiling sufficiently long time series that are systematic and can be reliably analysed. Chronosequences provide one popular solution, and several studies have assessed the importance of temporal context on lichen functional traits and the response to disturbance. For example, Giordani et al. [169] analysed the functional richness of epilithic lichen communities in dry grasslands subject to fire, showing that while community function was only slightly altered in relation to the time elapsed, it was reduced considerably as the frequency of fires increased. In the same way, Concostrina-Zubiri et al. [78,170,171] examined the effect of grazing on biological soil crust communities and recovery after livestock exclusion in the semiarid grasslands of Central Mexico as well as for Mediterranean cork-oak woodlands. They found that differences in grazing impact, and time of recovery from grazing, both resulted in slight shifts in species richness [78], but with important changes in species composition and cover which may affect ecosystem function. Additional results indicated that biocrust functional groups could be used as indicators of the disturbance-recovery processes [78]. The authors also noted that changes in functional composition along these gradients influence different ecosystem processes (see Effect Traits, above).

Aside from chronosequence work, direct monitoring data is limited, but has been applied notably in climate change impact studies. Van Herk et al. [172] found evidence that recent change in the lichen flora of the Netherlands was attributable to an increase in temperature. Compositional shifts that had occurred over the last 22 years were considered in relation to species biogeography, to show that arctic-alpine/boreo-montane lichens appeared to be declining, with (sub)tropical lichens increasing. Linking through to traits, Aptroot & van Herk [173] then showed that lichens responding to climate change in Western Europe, particularly epiphyte species that were increasing in forests, tended to contain *Trentepohlia* as the photobiont, in addition to having a southern distribution. In another global change study, epiphytic lichen communities were assessed over fifteen years in a semiarid region of Portugal [174], with functional diversity included as one of several metrics tested. Compositional changes were shown to be mediated through the response of lichen functional diversity, unrelated to species richness, and principally observed as the sensitivity of photobiont, growth form and size, to the number of days with relative humidity greater than 95%.

### 4.4. Interactions Across Scales

We conclude this section with the emphasis that variables act simultaneously across different spatial and temporal scales to affect lichens. With respect to global change, point-source responses, such as the presence of nitrophytic lichens in response to cattle grazing, could be relatively local, while climate change—although having local effects—is ultimately a global driver. There may be exceptional cases in which local drivers can lead to global responses, as in the case of radionuclide bioaccumulation [175] following nuclear accidents, while, conversely, large-scale drivers could cause local effects such as the consequences of isolation on individual populations following massive deforestation. Some lichen traits that are currently widely used in ecological studies, such as growth form, photobiont type, reproductive mode and production of metabolites, have been identified as relevant to broad-scale drivers such as macroclimatic conditions, pollution and disturbance gradients, etc. [34,40,176,177]. However, additional work is needed to understand the added value of more expensive to measure physiological traits (e.g., chlorophyll a content, water holding capacity, nutrient content and isotopes) and how these might correlate with or emerge from within broader traits, while responding to fine-scale drivers (e.g., microclimatic conditions) or, conversely, being more relevant as effect traits and useful to assess the impact of community structure on ecosystem function and services [39].

Although an explicit identification of the scale (i.e., biological, spatial or temporal) is necessary in analysis of response traits, the inter-linked consequences for ecosystem functioning remains a knowledge gap. There may be interactions between response and effect across scales. These cross-scale interactions have been observed for example by studying the functional over-redundancy and vulnerability of lichen communities in the Mediterranean environment [178]. Functional over-redundancy increased with spatial scale (creating ecosystem resilience), and functional vulnerability decreased with cross-scale interactions; increased warming and climatic extremes caused trait clustering into a small number of resistant (stress-tolerant) functional entities. Trait variation operating across levels of biological organisation may be especially relevant for the ecological dynamics of trophic systems. For instance, variability of lichen traits can mediate ecosystem processes relating to decomposition and nutrient cycling through an effect on invertebrates. In this context, studies have highlighted the between-scale effects of lichen secondary metabolites, and growth forms linked to thallus physiology, affecting invertebrate community composition [45,179,180,181]. Moreover, changes in invertebrate community composition may then operate as a feedback effect on the dispersal, establishment and survival of some lichen species through endozoochory or exozoochory processes [182,183,184,185,186].

## 5. Conclusions and Recommendations

Our review allows us to identify several key considerations for further work.

First, we highlight the speed of recent developments in trait-based studies. Methodological choices need to reflect a trend towards trait-based studies becoming more integrative, for example coupling traits with phylogeny to merge evolutionary and ecological processes. Although there may not be one correct analytical solution to solve a given hypothesis, it is nevertheless important to be clear about a method choice and its caveats. Consider the problem of trait combination and interactions. At the simplest level, one might use multivariate ordination, such as principal components analysis to summarise phenotypes as the covariation across a set of traits, and to analyse complex phenotypes as ordination axis, though this example would assume a linear correlative relationship in trait variation. This could be generalised through the use of non-linear multivariate methods, such as hierarchical agglomerative clustering or nonmetric multidimensional scaling (see [187,188]), while novel statistical approaches such as multivariate tree boosting [189,190] can handle interactions among multivariate response and predictor variables, though in the latter case limited to considering a small number of interactions at each stage of model building. Methods that allow greater flexibility in relationships among traits (e.g., nonmetric multidimensional scaling), have been combined with a non-linear interpretation of ordination space (e.g., nonparametric multiplicative regression), to understand how complex phenotypes fit into different portions of environmental space [191,192]. Similarly, Fourth Corner and RLQ Analysis [193,194,195] have been used to assess the relationship between community structure, functional traits and the environment, and extensions are now making it possible to account for phylogenetic signal in these types of approach [195,196]. Likewise, advanced statistics such as MCMCglmms make it possible to explore the interaction between environments and functional traits, while using the phylogenetic signal as a random effect.

Second, it is important that statistical methods, no matter how powerful, still draw from the selection of traits that are defensibly functional, with the danger that the addition of spurious—functionally unimportant—traits into multivariate analysis could remain hidden while confounding interpretation. It is therefore important that trait analyses across various scales (Figure 3) remain as supported as possible by evidence from experimental biology. This evidence is rapidly advancing with respect to key traits such as morphology [54,197], photobionts [25,198], reproductive mode [199], etc. Experimental evidence for the functionality of traits, allows them to be confidently included within integrative statistical models that can then more reliably test the value of these traits across the wider environment, and with respect to phylogeny.

Third, conclusions about traits are robust if they stand on multiple pillars of support: (i) They are consistent with field observation, (ii) They align with the results of experimentation, such as traits that are shown to have physiological consequences, and (iii) They show consistency with previously published analytical studies. However, this should not preclude novelty. Many morphological and metabolic characters routinely used in lichen taxonomy are still poorly quantified with regard to their precise functional roles. Few ecological papers include traits such as the presence or density and length of, for example, hairs [200], the types and thickness of cortex and medulla [201], subtypes and density of pseudocyphellae, or thallus texture, e.g., the subgelatinous texture in aquatic and subaquatic lichens [202], width and density of cracks in the thallus, etc. Studies are therefore needed that explore covariation between morpho-anatomical traits (e.g., growth form, size, number of laciniae, surface/volume ratio, cortical characteristic, anatomical structure of the thallus) and quantitative physiological data (e.g., FV/FM, chlorophyll a content, MDA, antioxidant activity, water storage), to better elucidate the interaction of lichens with specific ecological factors such as light, water, salts or herbivore pressure [38,59,200,203,204,205]. Novel insights could be generated by bringing new technologies to bear on lichen trait-based studies. High-throughput pipelines for the characterisation of lichen metabolites and the resulting reference libraries based on MS/MS spectra are quickly enlarging the resource for identification and quantification of metabolites, but they also indicate that older catalogues and widespread bibliographic references were omitting important metabolites even in seemingly well-known lichens such as *Hypogymnia physodes* [206]. The potential of neglected metabolite groups to improve our understanding in the functional role of metabolite mixtures has not been fully explored. Furthermore, the focus on metabolite profiling remains mostly on acetone soluble substances. Water soluble metabolites (e.g., mycosporines) could be important traits for otherwise inexplicable ecological performance of lichens that lack the better known and typical ‘lichen substances’ [207]. These developments need the support of a shared infrastructure to link morpho-anatomical and physiological data for a wide range of lichens from different geographic and climatic regions. Even for morpho-anatomical traits, the current data repositories for lichen-fungi lack direct access to original publications from which the data were aggregated and thus do not yet provide a centralised resource that includes a regional breakdown and comparison of trait variation across different geographical areas and scales. This goal could be efficiently achieved by modification of existing resources, or else through the establishment of a new infrastructure.

Fourth, it is established that lichens are involved in some key ecosystem processes though we know less about how these processes are affected by individual traits. We can therefore recommend screening experiments across large numbers of lichens with contrasting traits to understand their ecosystem effects [33,208]. Lichens are easy to transplant and it is therefore feasible to manipulate lichen communities. Transplantation studies have been used and should be used to evaluate how traits impact ecosystem functioning such as soil surface energy balance and lichen associated invertebrates across trophic levels [79,102]. Removal experiments with plants have likewise proved to be an efficient avenue in exploring relationships between functional groups and ecosystem processes [209,210,211]. However, despite lichens being a strong candidate for such experiments, this remains an unexplored opportunity that needs to be developed. Recent plant studies have demonstrated that combined response-effect trait frameworks can be powerful predictors of ecosystem function, with empirical tests having been carried out to evaluate response and effect relationships and roles [212]. However, this response-effect framework has hardly been used in lichens and would represent a considerable analytical development. Although we have addressed regulating and supporting services here, the link between response and effect traits could be widened to provisioning services considering lichen metabolites are also of interest in bioprospecting, with materials useful in cosmetics and pharmacy [213], as well as with respect to cultural services [214].

## Figures and Tables

**Table 1:** A glossary of terminology used to classify trait-based approaches.

Trait Classification	Explanation
Response trait	A phenotypic attribute that links to fitness differences (rates of establishment, survival, reproduction), affecting the lichen response to the environment
Effect trait	A phenotypic attribute that affects the lichen role in the ecosystem, and ecosystem functioning and services
Soft trait	An easily measured (often categorical) trait; such a trait may nevertheless be cost effective and provide high explanatory power
Hard trait	A hard to measure (often quantitative trait); these terms (soft and hard) are subjective and used differently across the ecological literature, e.g., a hard trait may sometimes be considered analogous to a proximal trait
Qualitative trait	A trait measured on a nominal or ordinal scale
Quantitative trait	A trait measured on an interval or ratio scale
Proximal trait	Borrowing from the language of niche theory, a trait that captures, relatively directly, the lichen physiological response or ecosystem effect
Distal trait	In contrast, a summary trait that is less directly related to the lichen environmental response or ecosystem effect
Compound trait	A trait (often distal) that integrates numerous proximal and direct effects, into a broader summary response to the environment or ecosystem effect
Intraspecific trait variability	The concept that variability in a trait—plasticity—can be a trait itself, creating potential for acclimation and affecting—through response/effect—the lichen niche

## Data Availability

No data were used in this study.

## Supplementary Materials

The following are available online at https://www.mdpi.com/article/10.3390/microorganisms9040766/s1, Table S1: An overview of studies that have aligned spatial and temporal scales, for different resolutions of biological organisation and the environmental response, Table S2: An overview of studies that have aligned spatial and temporal scales, for different resolutions of biological organisation and the ecosystem process.
