# Supplementary material for: Functional Traits in Lichen Ecology: A Review of Challenge and Opportunity
Source: Microorganisms. 2021 Apr 6;9(4):766. doi: 10.3390/microorganisms9040766 (PMC8067525; doi:10.3390/microorganisms9040766)
Supplement: Supplementary file 1 [file microorganisms-09-00766-s001.pdf]

microorganisms

Table S1: An overview of studies that have aligned spatial and temporal scales, for different resolutions of biological organisation and the environmental response that has been the focus for response trait analysis; cf. Figure 3. Spatial scales are those defined by Pearson & Dawson (2003): Global > 10000 km, Continental 2000 - 10000 km, Regional 200 - 2000 km, Landscape 10 - 200 km, Local 1 - 10 km, Site 10 - 1000 m, Micro < 10 m.

Spatial scale	Temporal scale	Biological scale	Environmental response	Reference
Global	Point measurement	Community	(1) Biogeochemistry; (3) Climate	[1]
Continental	Long-term	Species	(3) Climate	[2]
Continental	Point measurement	Community	(1) Biogeochemistry; (3) Climate	[3, 4]
Continental	Point measurement	Species	(1) Biogeochemistry; (3) Climate	[5]
Regional	Long-term	Community	(4) Fire	[6]
Regional	Long-term	Species	(3) Climate	[7]
Regional	Point measurement	Community	(1) Biogeochemistry; (2) Biotic interactions; (3) Climate; (5) Forest structure; (6) Geomorphology	[8, 9, 10, 11, 12]
Regional	Point measurement	Population	(2) Biotic interactions; (3) Climate; (5) Forest structure	[13, 14]
Regional	Point measurement	Species	(3) Climate	[15]
Landscape	Point measurement	Community	(1) Biogeochemistry; (2) Biotic interactions; (3) Climate; (4) Fire; (5) Forest structure; (6) Geomorphology; (7) Radiation; (8) Soil properties	[16, 17, 18, 19, 20, 21, 22, 23, 24]
Landscape	Point measurement	Population	(5) Forest structure; (7) Radiation	[25, 26]
Local	Long-term	Community	(1) Biogeochemistry; (3) Climate; (4) Fire	[27, 28, 29]
Local	Short-term	Species	(1) Biogeochemistry; (3) Climate	[30]
Local	Point measurement	Community	(1) Biogeochemistry; (3) Climate; (5) Forest structure; (6) Geomorphology	[31, 32, 33, 34, 35]
Local	Point measurement	Species	(3) Climate; (5) Forest structure	[36]
Site	Long-term	Community	(2) Biotic interactions; (3) Climate	[37, 38]
Site	Short-term	Population	(7) Radiation	[39]
Site	Short-term	Species	(1) Biogeochemistry	[40, 41, 42]
Site	Point measurement	Community	(1) Biogeochemistry; (3) Climate; (5) Forest structure; (7) Radiation; (8) Soil properties	[43, 44, 45, 46]
Site	Point measurement	Species	(1) Biogeochemistry	[47]
Micro	Point measurement	Population	(2) Biotic interactions; (3) Climate; (5) Forest structure	[13, 14]

1. Matos, P.; Geiser, L.; Hardman, A.; Glavich, D.; Pinho, P.; Nunes, A.; Soares, A.M.V.M.; Branquinho, C. Tracking global change using lichen diversity: towards a global-scale ecological indicator. *Methods Ecol. Evol.* **2017,** *8,* 788–798. DOI: 10.1111/2041-210X.12712
2. Sancho, L.G.; Allan Green, T.; Pintado, A. Slowest to fastest: extreme range in lichen growth rates supports their use as an indicator of climate change in Antarctica. *Flora* **2007**, *202*, 667–673. DOI: 10.1016/j.flora.2007.05.005
3. Giordani, P.; Calatayud, V.; Stofer, S.; Seidling, W.; Granko, O.; Fischer, R. Detecting the nitrogen critical loads on European forests by means of epiphytic lichens a signal-to-noise evaluation. *For. Ecol. Manag.* **2014,** *311*, 29–40. DOI: 10.1016/j.foreco.2013.05.048
4. Hurtado, P.; Prieto, M.; Aragón, G.; De Bello, F.; Martínez, I. Intraspecific variability drives functional changes in lichen epiphytic communities across Europe. *Ecology* **2020**, *101(6)*, e03017. DOI: 10.1002/ecy.3017
5. Hurtado, P.; Prieto, M.; Martínez-Vilalta, J.; Giordani, P.; Aragón, G.; López-Angulo, J.; Košuthová, A.; Merinero, S.; Díaz-Peña, E.M.; Rosas, T.; Benesperi, R.; Bianchi, E.; Grube, M.; Mayrhofer, H.; Nascimbene, J.; Wedin, M.; Westberg, M.; Martínez, I. Disentangling functional trait variation and covariation in epiphytic lichens along a continent-wide latitudinal gradient. *Proc. Royal Soc. B.* **2020,** *287,* 20192862. DOI: 10.1098/rspb.2019.2862
6. Miller, D.D.; Ota, Y.; Sumaila, U.R.; Cisneros-Montemayor, A.M.; Cheung, W.W.L. Adaptation strategies to climate change in marine systems. *Glob. Change Biol.* **2018,** *24*, e1–e14. DOI: 10.1111/gcb.13829
7. Nascimbene, J.; Casazza, G.; Benesperi, R.; Catalano, I.; Cataldo, D.; Grillo, M.; Isocrono, D.; Matteucci, E.; Ongaro, S.; Potenza, G.; Puntillo, D.; Ravera, S.; Zedda, L.; Giordani, P. Climate change fosters the decline of epiphytic *Lobaria* species in Italy. *Biol. Conserv.* **2016,** *201,* 377–384. DOI: 10.1016/j.biocon.2016.08.003
8. Giordani, P.; Brunialti, G.; Bacaro, G.; Nascimbene, J. Functional traits of epiphytic lichens as potential indicators of environmental conditions in forest ecosystems. *Ecol. Indic.* **2012,** *18,* 413–420. DOI: 10.1016/j.ecolind.2011.12.006

9. Hurtado, P., Prieto, M.; Aragón, G.; Escudero, A.; Martínez, I. Critical predictors of functional, phylogenetic and taxonomic diversity are geographically structured in lichen epiphytic communities. *J. Ecol.* **2019**, *107*, 2303–2316. DOI: 10.1111/1365-2745.13189
10. Marini, L.; Nascimbene, J.; Nimis, P.L. Large-scale patterns of epiphytic lichen species richness: Photobiont-dependent response to climate and forest structure. *Sci. Total Environ.* **2011**, *409*, 4381–4386. DOI: 10.1016/j.scitotenv.2011.07.010
11. Matos, P.; Pinho, P.; Aragón, G.; Martínez, I.; Nunes, A.; Soares, A.M.V.M.; Branquinho, C. Lichen traits responding to aridity. *J. Ecol.* **2015**, *103*, 451–458. DOI: 10.1111/1365-2745.12364
12. Stanton, D.E.; Chávez, J.H.; Villegas, L.; Villasante, F.; Armesto, J.; Hedin, L.O.; Horn, H. Epiphytes improve host plant water use by microenvironment modification. *Funct. Ecol.* **2014**, *28*, 1274–1283. DOI: 10.1111/1365-2435.12249
13. Benesperi, R.; Nascimbene, J.; Lazzaro, L.; Bianchi, E.; Tepsich, A.; Longinotti, S.; Giordani, P. Successful conservation of the endangered forest lichen *Lobaria pulmonaria* requires knowledge of fine-scale population structure. *Fungal Ecol.* **2018**, *33*, 65–71. DOI: 10.1016/j.funeco.2018.01.006
14. Merinero, S.; Rubio-Salcedo, M.; Aragón, G.; Martínez, I. Environmental factors that drive the distribution and abundance of a threatened cyanolichen in Southern Europe: A multi-scale approach. *Am. J. Bot.* **2014**, *101*, 1876–1885. DOI: 10.3732/ajb.1400077
15. Eriksson, A.; Gauslaa, Y.; Palmqvist, K.; Ekström, M.; Esseen, P.A. Morphology drives water storage traits in the globally widespread lichen genus *Usnea. Fungal Ecol.* **2018**, *35*, 51–61. DOI: 10.1016/j.funeco.2018.06.007
16. Asplund, J.; Wardle, D.A. Within-species variability is the main driver of community-level responses of traits of epiphytes across a long-term chronosequence. *Funct. Ecol.* **2014**, *28*, 1513–1522. DOI: 10.1111/1365-2435.12278
17. Benítez, A.; Aragón, G.; González, Y.; Prieto, M. Functional traits of epiphytic lichens in response to forest disturbance and as predictors of total richness and diversity. *Ecol. Indic.* **2018**, *86*, 18–26. DOI: 10.1016/j.ecolind.2017.12.021
18. Bässler, C.; Cadotte, M.W.; Beudert, B.; Heibl, C.; Blaschke, M.; Bradtka, J.H.; Langbehn, T.; Werth, S.; Müller, J. Contrasting patterns of lichen functional diversity and species richness across an elevation gradient. *Ecography* **2016**, *39*, 689–698. DOI:10.1111/ecog.01789
19. Degtjarenko, P.; Matos, P.; Marmor, L.; Branquinho, C.; Randlane, T. Functional traits of epiphytic lichens respond to alkaline dust pollution. *Fungal Ecol.* **2018**, *36*, 81–88. DOI: 10.1016/j.funeco.2018.08.006
20. Ellis, C.J.; Coppins, B.J. Contrasting functional traits maintain lichen epiphyte diversity in response to climate and autogenic succession. *J. Biogeogr.* **2006**, *33*, 1643–1656. DOI: 10.1111/j.1365-2699.2006.01522.x
21. Giordani, P.; Malaspina, P.; Benesperi, R.; Incerti, G.; Nascimbene, J. Functional over-redundancy and vulnerability of lichen communities decouple across spatial scales and environmental severity. *Sci. Total Environ*. **2019**, *666*, 22–30. DOI: 10.1016/j.scitotenv.2019.02.187
22. Koch, N.M.; Matos, P.; Branquinho, C.; Pinho, P.; Lucheta, F.; de Azevedo Martins, S.M.; Vargas, V.M.F. Selecting lichen functional traits as ecological indicators of the effects of urban environment. *Sci. Total Environ.* **2019**, *654*, 705–713. DOI: 10.1016/j.scitotenv.2018.11.107
23. Mallen-Cooper, M.; Eldridge, D.J.; Delgado-Baquerizo, M. Livestock grazing and aridity reduce the functional diversity of biocrusts. *Plant Soil* **2018**, *429*, 175–185. DOI: 10.1007/s11104-017-3388-5
24. Nelson, P.R.; McCune, B.; Roland, C.; Stehn, S. Non-parametric methods reveal nonlinear functional trait variation of lichens along environmental and fire age gradients. *J. Veg. Sci.* **2015**, *26*, 1–18. DOI: 10.1111/jvs.12286
25. Gauslaa, Y.; Coxson, D. Interspecific and intraspecific variations in water storage in epiphytic old forest foliose lichens. *Botany* **2011**, *89*, 787–798. DOI: 10.1139/B11-070
26. Rubio-Salcedo, M.; Merinero, S.; Martínez, I. Tree species and microhabitat influence the population structure of the epiphytic lichen *Lobaria pulmonaria*. *Fungal Ecol.* **2015**, *18*, 1–9. DOI: 10.1016/j.funeco.2015.08.002
27. Geiser, L.H.; Nelson, P.R.; Jovan, S.E.; Root, H.T.; Clark, C.M. Assessing ecological risks from atmospheric deposition of nitrogen and sulfur to US forests using epiphytic macrolichens. *Diversity* **2019**, *11*, 87. DOI: 10.3390/d11060087
28. Giordani, P.; Brunialti, G.; Calderisi, M.; Malaspina, P.; Frati, L. Beta diversity and similarity of lichen communities as a sign of the times. *Lichenologist* **2018**, *50*, 371–383. DOI: 10.1017/S0024282918000221
29. Giordani, P.; Rizzi, G.; Caselli, A.; Modenesi, P.; Malaspina, P.; Mariotti, M.G. Fire affects the functional diversity of epilithic lichen communities. *Fungal Ecol.* **2016**, *20*, 49–55. DOI: 10.1016/j.funeco.2015.11.003
30. Tretiach, M.; Piccotto, M.; Baruffo, L. Effects of ambient NO_x on chlorophyll a fluorescence in transplanted *Flavoparmelia caperata* (Lichen). *Environ. Sci. Technol.* **2007**, *41*, 2978–2984. DOI: 10.1021/es061575k
31. Giordani, P.; Incerti, G.; Rizzi, G.; Rellini, I.; Nimis, P.L.; Modenesi, P. Functional traits of cryptogams in Mediterranean ecosystems are driven by water, light and substrate interactions. *J. Veg. Sci.* **2014**, *25*, 778–792. DOI: 10.1111/jvs.12119

32. Munzi, S.; Correia, O.; Silva, P.; Lopes, N.; Freitas, C.; Branquinho, C.; Pinho, P. Lichens as ecological indicators in urban areas: beyond the effects of pollutants. *J. Appl. Ecol.* **2014,** *51*, 1750–1757. DOI: 10.1111/1365-2664.12304
33. Pinho, P.; Dias, T.; Cruz, C.; Sim Tang, Y.; Sutton, M.A.; Martins-Loução, M.-A.; Máguas, C.; Branquinho, C. Using lichen functional diversity to assess the effects of atmospheric ammonia in Mediterranean woodlands. *J. Appl. Ecol.* **2011,** *48*, 1107–1116. DOI: 10.1111/j.1365-2664.2011.02033.x
34. Rocha, B.; Pinho, P.; Branquinho, C.; Boieiro, M.; Matos, P. Bringing the concept of ammonia critical levels into managing cork-oak woodland for conservation. *For. Ecol. Manag.* **2019,** *453,* 117566. DOI: 10.1016/j.foreco.2019.117566
35. Roos, R.E.; van Zuijlen, K.; Birkemoe, T.; Klanderud, K.; Lang, S.I.; Bokhorst, S.; Wardle, D.A.; Asplund, J. Contrasting drivers of community-level trait variation for vascular plants, lichens and bryophytes across an elevational gradient. *Funct. Ecol.* **2019,** *33*, 2430–2446. DOI: 10.1111/1365-2435.13454
36. Pardow, A.; Hartard, B.; Lakatos, M. Morphological, photosynthetic and water relations traits underpin the contrasting success of two tropical lichen groups at the interior and edge of forest fragments. *AoB PLANTS* **2010,** *plq004*. DOI: 10.1093/aobpla/plq004
37. Ladrón de Guevara, M.; Gozalo, B.; Raggio, J.; Lafuente, A.; Prieto, M.; Maestre, F.T. Warming reduces the cover, richness and evenness of lichen-dominated biocrusts but promotes moss growth: insights from an 8 yr experiment. *New Phytol.* **2018,** *220,* 811–823. DOI: 10.1111/nph.15000
38. Pastore, A.I.; Prather, C.M.; Gornish, E.S.; Ryan, W.H.; Ellis, R.D.; Miller, T.E. Testing the competition–colonization trade-off with a 32-year study of a saxicolous lichen community. *Ecology* **2014,** *95,* 306–315. DOI: 10.1890/13-0253.1
39. Gauslaa, Y.; McEvoy, M. Seasonal changes in solar radiation drive acclimation of the sun-screening compound parietin in the lichen *Xanthoria parietina*. *Basic Appl. Ecol.* **2005,** *6,* 75–82. DOI: 10.1016/j.baae.2004.10.003
40. Casale, M.; Bagnasco, L.; Giordani, P.; Mariotti, M.G.; Malaspina, P. NIR spectroscopy as a tool for discriminating between lichens exposed to air pollution. *Chemosphere* **2015,** *134,* 355–360. DOI: 10.1016/j.chemosphere.2015.03.095
41. Munzi, S.; Cruz, C.; Branquinho, C.; Pinho, P.; Leith, I.D.; Sheppard, L.J. Can ammonia tolerance amongst lichen functional groups be explained by physiological responses? *Environ. Pollut.* **2014,** *187,* 206–209. DOI: 10.1016/j.envpol.2014.01.009
42. Paoli, L.; Pirintsos, S.A.; Kotzabasis, K.; Pisani, T.; Navakoudis, E.; Loppi, S. Effects of ammonia from livestock farming on lichen photosynthesis. *Environ. Pollut.* **2010,** *158,* 2258–2265. DOI: 10.1016/j.envpol.2010.02.008
43. Baldauf, S.; Ladrón de Guevara, M.; Maestre, F.T.; Tietjen, B. Soil moisture dynamics under two rainfall frequency treatments drive early spring CO_2 gas exchange of lichen-dominated biocrusts in central Spain. *PeerJ* **2018,** *6,* e5904. DOI: 10.7717/peerj.5904
44. Di Nuzzo, L.*; Vallese, C.*; Benesperi, R.; Giordani, P.; Chiarucci, A.; Di Cecco, V.; Di Martino, L.; Di Musciano, M.; Gheza, G.; Lelli, C.; Spitale, D.; Nascimbene, J. Contrasting multitaxon responses to climate change in Mediterranean mountains. *Sci. Rep.* In press. [*Join first authors]
45. Pinho, P.; Theobald, M.R.; Dias, T.; Tang, Y.S.; Cruz, C.; Martins-Loução, M.A.; Máguas, C.; Sutton, M.; Branquinho, C. Critical loads of nitrogen deposition and critical levels of atmospheric ammonia for semi-natural Mediterranean evergreen woodlands. *Biogeosciences* **2012,** *9,* 1205–1215. DOI: 10.5194/bg-9-1205-2012
46. Vieira, J.; Matos, P.; Mexia, T.; Silva, P.; Lopes, N.; Freitas, C.; Correia, O.; Santos-Reis, M.; Branquinho, C.; Pinho, P. Green spaces are not all the same for the provision of air purification and climate regulation services: The case of urban parks. *Environ. Res.* **2018,** *160,* 306–313. DOI: 10.1016/j.envres.2017.10.006
47. Malaspina, P.; Modenesi, P.; Giordani, P. Physiological response of two varieties of the lichen *Pseudevernia furfuracea* to atmospheric pollution. *Ecol. Indic.* **2018,** *86,* 27–34. DOI: 10.1016/j.ecolind.2017.12.028

Table S2: An overview of studies that have aligned spatial and temporal scales, for different resolutions of biological organisation and the ecosystem process that has been the focus for effect trait analysis; cf. Figure 3. Spatial scales are those defined by Pearson & Dawson (2003): Global > 10000 km, Continental 2000 - 10000 km, Regional 200 - 2000 km, Landscape 10 - 200 km, Local 1 - 10 km, Site 10 - 1000 m, Micro < 10 m.

Spatial scale	Temporal scale	Biological scale	Ecosystem process	Reference
Global	Point measurement	Community	(b) Biogeochemistry; (d) Canopy hydrology	[1, 2, 3]
Regional	Long-term	Community	(b) Biogeochemistry; (c) Biotic interactions; (f) Decomposition	[4, 5]
Regional	Point measurement	Community	(b) Biogeochemistry; (d) Canopy hydrology; (e) Climate regulation	[6, 7]
Landscape	Point measurement	Species	(f) Decomposition; (h) Lichenivory	[8]
Landscape-Local	Point measurement	Community	(a) Air purification	[9]
Local	Long-term	Community	(d) Canopy hydrology	[10]
Local	Long-term	Species	(b) Biogeochemistry; (d) Canopy hydrology	[11]
Local	Short-term	Species	(g) Invertebrate communities	[12]
Local	Point measurement	Community	(b) Biogeochemistry; (f) Decomposition; (i) Soil insulation	[6, 13, 14, 15]
Local	Point measurement	Species	(b) Biogeochemistry; (f) Decomposition; (g) Invertebrate communities	[15, 16]
Site	Long-term	Community	(b) Biogeochemistry	[17]
Site	Short-term	Species	(a) Air purification; (e) Climate regulation	[18]
Site	Point measurement	Community	(c) Biotic interactions	[19]
Site	Point measurement	Species	(c) Biotic interactions; (i) Soil insulation	[20]
Site-Micro	Long-term	Species	(e) Climate regulation; (f) Decomposition	[15]
Micro	Short-term	Species	(i) Soil insulation	[21]

1. Porada, P.; Van Stan, J.T.; Kleidon, A. Significant contribution of non-vascular vegetation to global rainfall interception. *Nat. Geosci.* **2018**, *11*, 563–567. DOI: 10.1038/s41561-018-0176-7
2. Porada, P.; Weber, B.; Elbert, W.; Pöschl, U.; Kleidon, A. Estimating impacts of lichens and bryophytes on global biogeochemical cycles. *Glob. Biogeochem. Cycles* **2014**, *28*, 71–85. DOI: 10.1002/2013GB004705
3. Porada, P.; Weber, B.; Elbert, W.; Pöschl, U.; Kleidon, A. Estimating global carbon uptake by lichens and bryophytes with a process-based model. *Biogeosciences* **2013**, *10*, 6989–7033. DOI: 10.5194/bg-10-6989-2013
4. Lang, S.I.; Cornelissen, J.H.C.; Klahn, T.; Logtestijn, R.S.P.V.; Broekman, R.; Schweikert, W.; Aerts, R. An experimental comparison of chemical traits and litter decomposition rates in a diverse range of subarctic bryophyte, lichen and vascular plant species. *J. Ecol.* **2009**, *97*, 886–900. DOI: 10.1111/j.1365-2745.2009.01538.x
5. Rickbeil, G.J.M.; Hermosilla, T.; Coops, N.C.; White, J.C.; Wulder, M.A. Estimating changes in lichen mat volume through time and related effects on barren ground caribou (*Rangifer tarandus groenlandicus*) movement. *PLOS ONE* **2017**, *12*, e0172669. DOI: 10.1371/journal.pone.0172669
6. Bowker, M.A.; Mau, R.L.; Maestre, F.T.; Escolar, C.; Castillo-Monroy, A.P. Functional profiles reveal unique ecological roles of various biological soil crust organisms. *Funct. Ecol.* **2011**, *25*, 787–795. DOI: 10.1111/j.1365-2435.2011.01835.x
7. Stanton, D.E.; Chávez, J.H.; Villegas, L.; Villasante, F.; Armesto, J.; Hedin, L.O.; Horn, H. Epiphytes improve host plant water use by microenvironment modification. *Funct. Ecol.* **2014**, *28*, 1274–1283. DOI: 10.1111/1365-2435.12249
8. Asplund, J.; Wardle, D.A. The impact of secondary compounds and functional characteristics on lichen palatability and decomposition. *J. Ecol.* **2013**, *101*, 689–700. DOI:10.1111/1365-2745.12075
9. Matos, P.; Vieira, J.; Rocha, B.; Branquinho, C.; Pinho, P. Modeling the provision of air-quality regulation ecosystem service provided by urban green spaces using lichens as ecological indicators. *Sci. Total Environ.* **2019**, *665*, 521–530. DOI: 10.1016/j.scitotenv.2019.02.023
10. Pypker, T.G.; Unsworth, M.H.; Bond, B.J. The role of epiphytes in rainfall interception by forests in the Pacific Northwest. I. Laboratory measurements of water storage. *Can. J. For. Res.* **2006**, *36*, 809–818. DOI: 10.1139/x05-298
11. Knops, J.M.; Nash III, T.H.; Schlesinger, W.H. The influence of epiphytic lichens on the nutrient cycling of an oak woodland. *Ecol. Monogr.* **1996**, *66*, 159–179. DOI: 10.2307/2963473
12. Asplund, J.; Bokhorst, S.; Kardol, P.; Wardle, D.A. Removal of secondary compounds increases invertebrate abundance in lichens. *Fungal Ecol.* **2015**, *18*, 18–25. DOI: 10.1016/j.funeco.2015.07.009
13. Aartsma, P.; Asplund, J.; Odland, A.; Reinhardt, S.; Renssen, H. Surface albedo of alpine lichen heaths and shrub vegetation. *Arct. Antarct. Alp. Res.* **2020**, *52*, 312–322. DOI: 10.1080/15230430.2020.1778890

14. Rocha, B.; Pinho, P.; Branquinho, C.; Boieiro, M.; Matos, P. Bringing the concept of ammonia critical levels into managing cork-oak woodland for conservation. *For. Ecol. Manag.* **2019,** *453,* 117566. DOI: 10.1016/j.foreco.2019.117566
15. van Zuijlen, K.; Roos, R.E.; Klanderud, K.; Lang, S.I.; Asplund, J. Mat-forming lichens affect microclimate and litter decomposition by different mechanisms. *Fungal Ecol.* **2020**, *44*, 100905. DOI: 10.1016/j.funeco.2019.100905
16. Bokhorst, S.; Asplund, J.; Kardol, P.; Wardle, D.A. Lichen physiological traits and growth forms affect communities of associated invertebrates. *Ecology* **2015**, *96*, 2394–2407. DOI: 10.1890/14-1030.1
17. Ladrón de Guevara, M.; Gozalo, B.; Raggio, J.; Lafuente, A.; Prieto, M.; Maestre, F.T. Warming reduces the cover, richness and evenness of lichen-dominated biocrusts but promotes moss growth: insights from an 8 yr experiment. *New Phytol.* **2018,** *220,* 811–823. DOI: 10.1111/nph.15000
18. Vieira, J.; Matos, P.; Mexia, T.; Silva, P.; Lopes, N.; Freitas, C.; Correia, O.; Santos-Reis, M.; Branquinho, C.; Pinho, P. Green spaces are not all the same for the provision of air purification and climate regulation services: The case of urban parks. *Environ. Res.* **2018,** *160,* 306–313. DOI: 10.1016/j.envres.2017.10.006
19. Maier, S.; Tamm, A.; Wu, D.; Caesar, J.; Grube, M.; Weber, B. Photoautotrophic organisms control microbial abundance, diversity, and physiology in different types of biological soil crusts. *ISME J.* **2018**, *12*, 1032–1046. DOI: 10.1038/s41396-018-0062-8
20. Nystuen, K.O.; Sundsdal, K.; Opedal, Ø.H.; Holien, H.; Strimbeck, G.R.; Graae, B.J. Lichens facilitate seedling recruitment in alpine heath. *J. Veg. Sci.* **2019**, *30*, 868–880. DOI: 10.1111/jvs.12773
21. Carter, N.E.A.; Viles, H.A. Lichen hotspots: Raised rock temperatures beneath *Verrucaria nigrescens* on limestone. *Geomorphology* **2004**, *62*, 1–16. DOI: 10.1016/j.geomorph.2004.02.001

Publisher's Note: MDPI stays neutral with regard to jurisdictional claims in published maps and institutional affiliations.

© 2021 by the authors. Submitted for possible open access publication under the terms and conditions of the Creative Commons Attribution (CC BY) license (http://creativecommons.org/licenses/by/4.0/).
